# Wearable Continuous Blood Pressure Monitoring Devices Based on Pulse Wave Transit Time and Pulse Arrival Time: A Review

**DOI:** 10.3390/ma16062133

**Published:** 2023-03-07

**Authors:** Zi-Bo Zhou, Tian-Rui Cui, Ding Li, Jin-Ming Jian, Zhen Li, Shou-Rui Ji, Xin Li, Jian-Dong Xu, Hou-Fang Liu, Yi Yang, Tian-Ling Ren

**Affiliations:** 1 School of Integrated Circuit, Tsinghua University, Beijing 100084, China; 2 Beijing National Research Center for Information Science and Technology (BNRist), Tsinghua University, Beijing 100084, China; 3 Center for Flexible Electronics Technology, Tsinghua University, Beijing 100084, China; 4 School of Mechanical, Electrical and Information Engineering, Shandong University, Weihai 264209, China

**Keywords:** pulse transit time, cuffless blood pressure, wearable devices, PPG, ECG

## Abstract

Continuous blood pressure (BP) monitoring is of great significance for the real-time monitoring and early prevention of cardiovascular diseases. Recently, wearable BP monitoring devices have made great progress in the development of daily BP monitoring because they adapt to long-term and high-comfort wear requirements. However, the research and development of wearable continuous BP monitoring devices still face great challenges such as obvious motion noise and slow dynamic response speeds. The pulse wave transit time method which is combined with photoplethysmography (PPG) waves and electrocardiogram (ECG) waves for continuous BP monitoring has received wide attention due to its advantages in terms of excellent dynamic response characteristics and high accuracy. Here, we review the recent state-of-art wearable continuous BP monitoring devices and related technology based on the pulse wave transit time; their measuring principles, design methods, preparation processes, and properties are analyzed in detail. In addition, the potential development directions and challenges of wearable continuous BP monitoring devices based on the pulse wave transit time method are discussed.

## 1. Introduction

Hypertension is a major factor in many cardiovascular diseases [1], and cardiovascular disease is the top global cause of death in the world [2]. According to the World Health Organization, hypertension is defined as systolic blood pressure (SBP) of more than 140 mmHg and diastolic blood pressure (DBP) of more than 90 mmHg [3]. Moreover, China’s latest hypertension clinical practice guideline, released in November 2022, suggested that patients with an SBP above 130 mmHg and a DBP above 80 mmHg are considered hypertensive [4]; this is 10 mmHg lower than the World Health Organization’s standard for judging hypertension. However, although China’s National Health Commission has not adjusted the criteria for judging hypertension, it may be a trend to update the criteria.

In recent years, the number of people with hypertension has risen continuously. The number of adults aged 30−79 with hypertension increased from 650 million to 1.28 billion all over the world [3]. For most people with hypertension, there are no symptoms before the onset, but the complications resulting from hypertension (such as cerebral hemorrhage, coronary heart disease, heart failure, etc.) contribute to 9.4 million deaths in the world every year [5]. Therefore, it is of great significance to treat hypertensive patients as early as possible.

**Figure 1:**
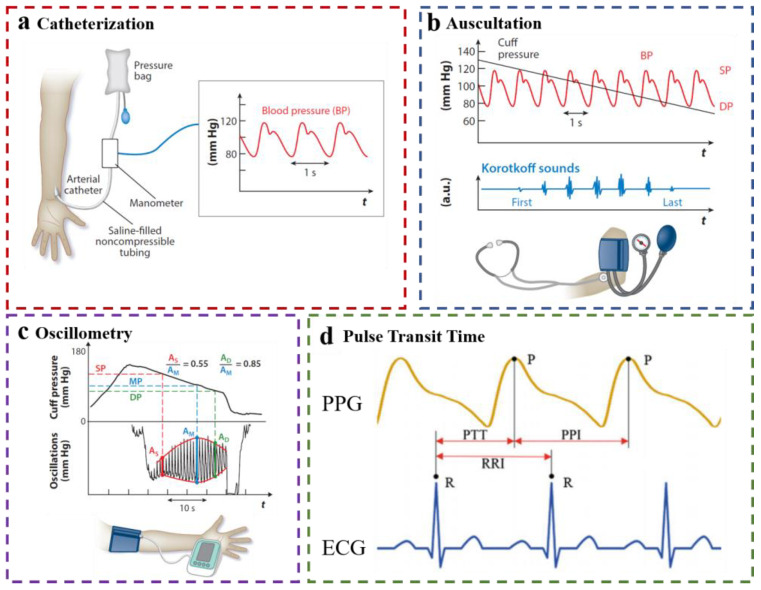
(**a**) Arterial catheterization method; (**b**) Cuff-based auscultation method; (**c**) Cuff-based oscillometry method; Reproduced with the permission of [27]. Copyright 2022, Annual Reviews. (**d**) PTT duration was determined by the time difference between the ECG R-peak and PPG systole peak [29,30,31,32,33]. Reproduced with the permission of [33]. Copyright 2019, Springer Nature Singapore Pte Ltd.

An accurate diagnosis including the blood pressure measuring should be made before treating the patient. Clinical blood pressure (BP) measurement methods are divided into invasive methods and non-invasive methods; the gold standard for invasive methods is the invasive catheterization method (Figure 1a), and the gold standard for noninvasive methods is the cuff-based method (Figure 1b,c). Invasive catheterization methods are commonly used in intensive care units (ICU); a catheter needs to be inserted into the patient’s major artery [6], which is the most accurate measurement method, but this invasive method of measuring BP requires an injection of anesthetic, which is feasible outside the hospital. Additionally, the invasive catheter method not only requires professional medical personnel to operate but also may cause potential risks to patients such as infection and vascular damage [7], making frequent use and continuous BP measurement unattainable. The cuff-based method is a noninvasive measurement often recommended by doctors; however, this method also has many disadvantages: the size of the cuff is inappropriate, repeated inflating and pressurizing may cause discomfort and even tissue damage to the patient, cuff-based devices are not easily portable, and the user cannot move during use, which can easily lead to the inconvenience of their use [8]. Above all, the two gold standard methods share the same drawback: both of them only provide a snapshot of dynamic BP readings rather than continuous BP throughout the day and night [9]. Intermittent measurement is easily affected by the external environment; for example, the patient’s physical exercise before detection, the mood during detection, and other factors will affect the accuracy of the clinical intermittent measurement of BP, thus causing white coat hypertension (a condition in which the patient’s BP is high when measured in a medical environment but normal in daily activities, which may be caused by the patient’s anxiety and nervousness during clinical examination [10]). With the development of technology, the traditional intermittent BP measurement is not suitable for long-term use and remote monitoring. Only by realizing daily continuous BP monitoring can the timely diagnosis and treatment of patients’ physical conditions be realized. However, most of the existing reviews about continuous non-invasive blood pressure focus on the blood pressure estimation algorithm and models. The device/sensor required by the PTT method is the foundation of the performance of the PTT-based measuring system, which determines the upper limit of the accuracy of the blood pressure estimation models. Therefore, this paper summarizes the devices required by the wearable cuffless BP monitoring method and hopes to help guide the design of future wearable BP monitoring systems to achieve practical continuous BP monitoring.

Recently, the wearable cuffless BP monitoring system has shown great potential in the field of continuous BP monitoring because it is suitable for home continuous BP self-measurement and remote monitoring [11]. There are a lot of cuffless methods; examples include acoustic [12], piezoelectric [13], bioimpedance [14], etc. The volume of ultrasound generators required by the acoustic method is large, and it is difficult to combine with a dynamic sensor [15]. The piezoelectric method has poor biological compatibility and is incompatible with the elasticity of the skin, which can affect the measurement accuracy and the comfort of the wearable device. The bioimpedance method uses the graphene electronic tattoo that is atomically thin and thick and has great biocompatibility [14]. However, the bioimpedance approach requires more graphene bioimpedance tattoos to be deployed on the wrist, which affects comfort and is difficult to be integrated into the modern wearable devices such as smartwatches and other devices. Among the existing methods, using pulse transit time (PTT) to estimate BP is a more promising continuous BP monitoring method [16]. Both signals used to calculate PTT can be obtained by noninvasive methods, which will not cause physical discomfort. The small size of the sensors used to obtain these two signals and their high biological compatibility not only ensures users’ comfort but also means they can be easily integrated into many modern wearable devices. For example, HUAWEI WATCH D [17] and Samsung Galaxy Watch 5 [18] have integrated the technology into their smart wristbands. ECG signals can be collected intermittently at the wrist, and PPG signals can be collected simultaneously at the wrist, which allows users to monitor their BP continuously for 24 h.

In recent years, a growing number of studies have used the PTT technique to estimate BP continuously. Wang et al. developed a viable portable noninvasive continuous BP monitoring device [19] to derive PPT by collecting the ECG signal of the head and the PPG signal of the brow bone, while correlation and regression analyses of BP values were performed to establish a PTT-based BP calculation model. Compared with the traditional noninvasive BP measurement method, the error was maintained within 5%. In addition to using PTT as a single indicator, the researchers also found that the accuracy of the BP calculation model could be improved, as other static biometric indicators such as age, sex, and body mass index (BMI) were added [20,21]. Wang et al. developed a multi-parameter BP calculation model [22] with the input of feature vectors composed of PTT, age, sex, and body mass index (BMI) as indicators and calculated the SBP and DBP with a mean error ± standard deviation of − 2.10 ± 7.07 mmHg and 0.04 ± 7.34 mmHg, and the accuracy of BP calculations has been improved to meet the requirements of the Association for the Advancement of Medical Instrumentation (AAMI). However, despite the current rapid progress in research on continuous BP monitoring devices based on PTT methods, there are still many problems because wearable monitoring sensors are susceptible to various noise sources and external disturbances [23], such as skin color, age, stress, motion artifacts, etc. Among them, the PPG sensor is the most sensitive to motion artifacts and has the greatest influence on the accuracy of the PPG signal. There are many analog filters and DSP methods for eliminating motion artifacts from distorted PPG signals, but they can only eliminate motion artifacts to a limited extent [24]. Therefore, eliminating motion artifacts in PPG signals is a major difficulty in developing continuous BP detection equipment in the future.

**Figure 2:**
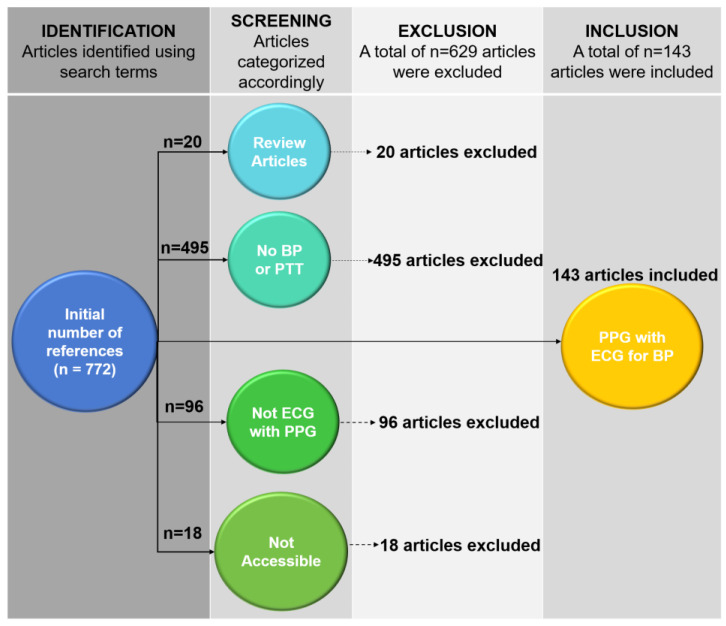
Flowchart of the methodology used to include 143 out of 772 published studies.

The literature search strategy of this review is as follows: the selected international databases include Web of Science, IEEE, PubMed, Scopus, and Chinese Science Citation Database. The search keywords for topics and titles included: (1) “blood pressure”, or “blood pressure estimation”, or “blood pressure measurement”, or “blood pressure monitoring”, or “bp”; (2) “photoplethysmogram”, or “photoplethysmography”, or “PPG”; (3) “electrocardiogram”, or “electrocardiography”, or “ECG”. Through the application of the above-mentioned keywords, the literature search returned 772 references. Then, the found research literature was further screened by applying such criteria as: (1) written in English; (2) peer-reviewed journal articles, excluding official governmental reports; (3) focus on the wearable continuous BP monitoring devices based on PTT. This finally left 143 studies, and we decided to cite 92 of these literature works after reading them (Figure 2).

To our knowledge, few kinds of literature comprehensively summarize PTT-based continuous BP monitoring devices at present. In 2020, W. Kaylie et al. reviewed the methodology and principle for estimating BP by combining PPG with other biological signals [25], such as the aortic valve, ECG, impedance plethysmography, etc. In 2021, S. Menish et al. summarized the research on the automatic detection arterial of hypertension based on ECG, HRV, PPG, and BCG signals [26] and discussed the use of the database, the performance of the machine learning model, and the deep learning model. In 2022, M. Ramakrishna et al. reviewed the methods of continuous non-invasive BP monitoring [27], various cuff methods and cuffless methods were summarized in the paper, and the concept, measurement position, and calibration model of the PTT method were summarized, but the devices and sensors required for PTT measurement were not mentioned. In 2022, B. Daniel et al. also reviewed the PTT method [28], but the content of the review focused on the BP estimation algorithm and calibration model. Therefore, the information summarized in this paper may help guide the design of future wearable BP monitoring systems to achieve practical continuous BP monitoring. In this review, we introduce the recent state-of-the-art advances in continuous BP monitoring devices based on PTT. The first part introduces the advantages and disadvantages of the two clinical gold standards for BP measurement, introduces the PTT method, which is more suitable for daily continuous BP monitoring, and cites several cases to prove the feasibility of the PTT method. The second part further reviews various PTT continuous BP monitoring devices. Their measuring principles, design methods, preparation processes, and properties are analyzed in detail. The third part introduces the possible development direction and possible challenges of the PTT method in the future. Finally, the future development prospect of wearable continuous BP monitoring devices is summarized from our perspective.

## 2. PTT-Based Devices for Continuous BP Monitoring

### 2.1. Definition of Pulse Transit Time (PTT) and Pulse Arrival Time (PAT)

Pulse transit time (PTT) is the time it takes for a pulse wave to travel between two places in the cardiovascular system, also known as the time it takes for a pulse wave to travel along a fixed arterial segment of length [34]. PTT is a powerful physiological parameter that has been widely used in various clinical applications, such as sleep quality monitoring [35], the evaluation of arterial stiffness [9], myocardial ischemia [36], etc. However, with the continuous development of research, more and more studies have demonstrated that changes in PTT can reliably track changes in BP and indirectly measure BP [37,38,39]. Generally, if the BP is higher, the blood will travel faster, and the PTT will be shorter over the same distance; if the BP is lower, the blood will travel slower, and the PTT will be longer for the same distance, which means that there is a conversion relationship between BP and PTT. Therefore, PTT becomes an indicator for estimating BP, and a linear or nonlinear regression model can be established according to PTT to calculate BP [9].

The definitions of Pulse Arrival Time (PAT) and PTT are similar, but there are also significant differences. PAT usually refers to the interval between the R peak of the ECG signal and the peak of the PPG signal, and PAT is the sum of the PTT and the pre-ejection time (PEP). PEP is the time between the electrical systole (ECG Q wave) and the initial opening of the aortic valve [40], and it is usually negligible and relatively constant. The R-wave is typically used instead of the Q-Wave [41]. In many references, researchers refer to PAT simply as PTT, such as in Figure 1d and Wang et al. [22].

Among various regression models, the linear regression model using only PTT is the simplest BP estimation model. In addition to the linear regression model based on PTT, there are many nonlinear regression models, such as reciprocal type [42], exponential type [43], logarithmic type [9], and so on. All of these models can complete the estimation of BP based on PTT, but there are differences in the calculation accuracy and computational complexity. In addition, PTT can also be combined with other physiological indicators (heart rate [44], age and gender [22], etc.) to form a multiple regression model, which can reflect human physiological information more comprehensively, thus improving the accuracy of continuous BP estimation. In actual research, appropriate physiological indicators and calculation models should be selected according to the characteristics of individuals.

### 2.2. Acquisition of PTT/PAT

PAT is the interval between the peak value of the R wave of the ECG signal and the peak value of the PPG signal; researchers usually calculate PAT by identifying these two peaks, and some researchers treat PAT as PTT. Therefore, the ECG and PPG signals must be obtained through the ECG sensor and the PPG sensor first. However, the quality of the ECG and PPG signals directly affects the accuracy of the PTT and, thus, the accuracy of the BP estimation model. Thus, the quality of the ECG sensor and PPG sensor is crucial, and the measuring principles, design methods, preparation processes, and properties of many ECG sensors and PPG sensors are discussed below to provide a reference and guidance for the design of future ECG sensors and PPG sensors. In addition, there are also other ways to calculate PAT; J. Sola et al. proposed a method for estimating PAT based only on the characteristic point of the pressure pulse waveform, without the ECG signal [45]. We can also estimate PTT instead of measuring it; J. Sola et al. established a lumped model of the segments of the arterial tree involved in Pulse Wave Velocity (PWV) measurements [46], so PTT can be estimated by the formula PTT = distance/PWV.

### 2.3. PPG Sensors

**Figure 3:**
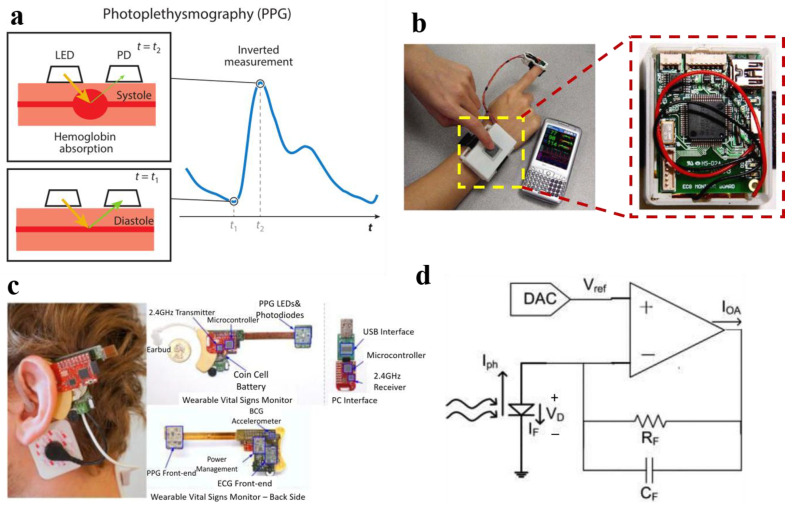
(**a**) PPG technology. Abbreviations: LED, light-emitting diode; PD, photodetector; Reproduced with the permission of [27]. Copyright 2022, Annual Reviews. (**b**) The wrist-worn device and phone display; Reproduced with the permission of [53]. Copyright 2010, IEEE. (**c**) Wearable vital signs monitor worn at the ear; (**d**) Static photocurrent subtraction circuit. Reproduced with the permission of [55]. Copyright 2012, IEEE.

PPG is a noninvasive optical measurement technique [23]; it uses the principle of light absorption in the blood to provide information for the monitoring of human physiological health. Here is the principle of PPG technology. First, a certain intensity of light is shone onto the body tissue (Figure 3a), which will absorb light. The transmitted or reflected light intensity will have a certain attenuation, and the muscle, bone, vein, and other connective tissue’s absorption of light is constant, but every heartbeat, contraction, and expansion of blood vessels will cause blood volume changes, resulting in a great change in the absorption of light. Second, the intensity of light transmitted or reflected from the skin tissue is measured [27], and after converting optical signals into electrical signals, DC signals (corresponding to the light absorption of muscles, bones, veins, and other connective tissues) and AC signals (corresponding to the light absorption of blood) can be extracted. The AC signal can reflect the change in blood volume, and the AC signal is known as the PPG signal.

PPG sensors are available in transmission and reflection modes. Reflective mode PPG sensors can be placed anywhere on the human skin for detection, so they are more commonly used in wearable devices. PPG sensors usually use green, red, or infrared light for detection, among which green light has better performance than other sources in detecting blood flow changes on the skin surface [47], so it is more suitable for PPG sensors.

The performance of the PPG sensor is limited by its motion sensitivity, and the quality of the signal is easily affected by motion artifacts. Back in 2001, Renevey et al. tried to suppress motion artifacts through nonlinear modeling [48]; they realized a robust estimation of the heart rate based on the physiological properties of the heart. The heart rate can even be accurately estimated under intense exercise, but they still cannot obtain high-quality PPG signals due to the influence of the motion artifact. Therefore, obtaining high-quality PPG signals under the influence of motion artifacts is still a largely unsolved problem.

In 2006, Park et al. proposed an unconstrained PAT monitoring system using a capacitively coupled signal measurement system and an air cushion with a system of balancing tubes in a chair [49]. The subject does not need to be in contact with the PPG probe or the ECG electrodes and is not constrained by the PPG and ECG sensors. The subject only needs to sit in a chair equipped with the test device to complete the PPT monitoring. However, the requirement of sitting in a chair limits the subject’s freedom of movement and does not meet the need for continuous daily monitoring.

In order to facilitate the user’s free movement, in 2008, Jung et al. developed a physiological signal detection system adapted to the wrist [50]. The system is composed of an analog part, a digital part, a Bluetooth module, and a battery worn on the wrist. The PPG sensor and ECG sensor were integrated into the same PCB with a size of 8 × 4 × 1.6 cm. ECG and PPG signals were digitized at a sampling rate of 1200 Hz and an 8-bit resolution, and the PTT could be accurately extracted and transmitted wirelessly to the PC when the subject was tested while the body was still. When the physiological signal data are transmitted to the PC terminal, various physiological indicators such as BP, blood oxygen, and heart rate can be calculated. However, the PC terminal is not portable, and the subjects cannot obtain their physiological information anytime and anywhere. With the development of information technology, more researchers no longer transmit data to PCs wirelessly but rather to mobile devices, such as personal digital assistants (PDA), wristwatches, smartphones, and so on.

In 2008, Espina et al. measured PPG signals using a finger-type version PPG sensor [51], sampled at a frequency of 200 Hz and obtained by the IEEE 802.15.4 standard for wireless data transmission. The clock synchronization of PPG and ECG signals was carried out through flooding time synchronization protocol and eight-point linear regression. A 2 s synchronization message interval was used to transmit data to the PDA and wristwatch, which realize a data alignment precision better than 100 μs [52]. Subjects can check their physiological status anytime and anywhere. However, the system is not compact, and it requires subjects to wear three devices at the same time; thus, both portability and comfort need to be improved. In the experiment, it was found that the PPG signal quality is good when the subject is stationary, but when the subject is walking, the PPG signal is seriously affected by the motion artifact, and it is usually unable to extract an effective PPG signal. In daily life, the users will not stay static for a long time but will be accompanied by walking, movement, and other behaviors. Therefore, optimizing the performance of wearable devices under dynamic conditions is a big challenge. In the future, it is necessary to study the influence of posture changes on the PPG signal and motion artifact correction algorithm to eliminate the influence of the motion artifact.

In 2010, Xu et al. developed a relatively compact PPT monitoring system and encapsulated the system in the shell of a watch (Figure 3b) [53]. The highly integrated system is convenient for daily carrying. It includes a TlMSP430F1611 microcontroller, a PPG and ECG amplifier circuit, Bluetooth, and other modules, which can extract an effective PPG signal and calculate PTT combined with the ECG signal. According to the simplified force motion model, the BP value within each heartbeat cycle can be calculated, and the data can be transmitted wirelessly to the smartphone. Subjects only need to wear one device to complete all measurements, which has good portability. However, the volume of the wearable device is still large, and it is not convenient for the subjects to sleep with the device. Therefore, the proposed device cannot achieve continuous BP monitoring at night. Designing and manufacturing more delicate and comfortable wearable devices is a major challenge for the future development of PTT wearable systems.

In addition to improving portability and integration, the studies of Espina et al. and Xu et al. used finger type PPG sensors to measure PPG signals (Espina et al. [51]; Xu et al. [53]). With the increase in finger clamping time, the discomfort of the subjects would also increase, and it might even cause damage to the fingers. Therefore, the unobtrusive use of the PPG sensor and more comfortable is a trend to optimize PPG sensors design.

In 2012, Franco et al. proposed a wearable wireless body sensor network (WBSN) based on the IEEE 802.15.4 standard [54], which included a PPG sensor node located on the forehead and an ECG sensor node located on the chest. The PPG sensor adopted a reflective forehead sensor from Nonin Medical, instead of it being located on the fingers, which eliminates the discomfort of installing sensors on the fingers. The PPG and ECG signals were synchronized at the MAC level using the hardware features provided by ATmega128RFA1. The accuracy of the synchronization was high, with errors ranging from −16 μs to 64 μs. The PAT can be accurately extracted, and the calculated BP meets the requirements of the AAMI standard (no more than 5 ± 8 mmHg). It improves the comfort of the wearable system while realizing continuous BP monitoring.

In 2012, Winokur et al. designed an ear-mounted vital signs monitor (Figure 3c) and developed a static photocurrent subtraction circuit for PPG signal monitoring (Figure 3d) [55] to limit the LED optical output power in the PPG sensor to between 0.027 and 0.068 lumens. The LED drive current in the PPG sensor was reduced, successfully solving the problem of a low voltage single power supply system which is difficult to provide a large LED drive current. Combined with the ECG signal, the BP could be accurately estimated, with a mean error of −0.07 mmHg and a standard deviation of 3.64 mmHg, respectively. Ear monitors are similar in appearance to hearing aids; they are concealed since the devices can be partially hidden by the hair and the ear, avoiding the defects of finger-type devices.

**Figure 4:**
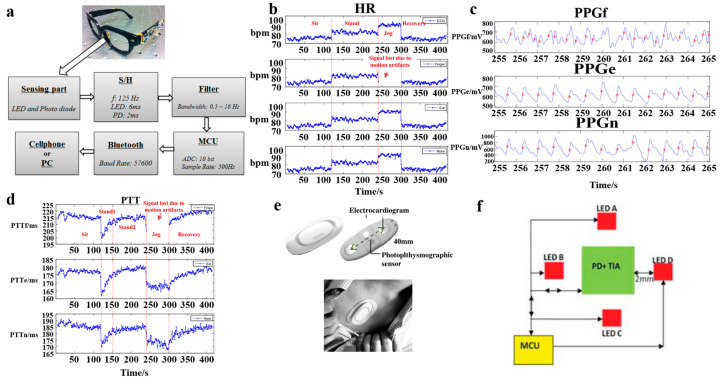
(**a**) The eyeglasses-based PPG measurement device; (**b**) The averaged HR; (**c**) PPGf, PPGe, and PPGnrch are PPG measured from the finger, ear, and nose, respectively; (**d**) The averaged PTT; Reproduced with the permission of [56]. Copyright 2012, IEEE. (**e**) Prototype of a wearable sensing device; Reproduced with the permission of [57]. Copyright 2013, IEEE. (**f**) Block diagram for the proposed 4-LED/one-photodiode PPG sensor. Reproduced with the permission of [44]. Copyright 2016, IEEE.

In 2012, Zheng et al. designed a glass-based PPG signal measurement device [56], with an optical sensor, a filter, a microcontroller unit (MCU, ATmega8), Bluetooth (WT12), and other modules mounted on the surface of the glasses. The near-infrared LED transmitter and photodiode (PD) on the nose bridge of the glasses are in direct contact with the skin to measure the PPG signal (Figure 4a). To verify the performance of the system, the system was compared with the finger-type and ear-mounted monitoring systems. The subjects’ heart rate (HR), PPG signal, and PTT were measured under the conditions of standing, sitting, and jogging, respectively (Figure 4b−d). Note that the finger detection system was most affected by motion noise, and the measured data can only be used when the subject is at rest. The proposed glass-based device not only measures the signal when the subject is at rest but also measures the PPG signal and the PTT of higher quality from the nose bridge when the subject is jogging, providing better performance than the finger-type detection system and the same performance as the ear-mounted monitoring system.

In 2013, Puke et al. developed a small physiological signal monitoring device to measure PTT over a short propagation distance [57]. The device incorporated an ECG sensor and a PPG sensor (Figure 4e). Two metal electrodes were placed 40 mm apart to detect the potential difference on the surface of the body to obtain the ECG signal. The reflective green-LED PPG sensor was placed between the electrodes, and a band-pass filter was implemented in the forward and backward directions to suppress the noise caused by body movement. The two signals were transmitted to the PC via Bluetooth. However, due to the short transmission distance, the extraction error of PTT was relatively high, and the estimated BP error was 5.87 mmHg to 9.41 mmHg. Although it failed to meet the requirements of AAMI, it was very close to the AAMI standard. This study proves the feasibility of measuring PTT at a relatively short transmission distance. In the future, the ECG and PPG sensors for the continuous BP monitoring device may no longer need to be located in different parts of the body but can be integrated for greater portability and comfort.

With the development of PPG technology, the estimation of motion artifacts is still a difficult problem to solve. PPG sensors usually contain an LED and a photodiode (PD). When the PPG sensor is working, the photodiode (PD) needs to receive the reflected light. Researchers have found that, in many cases, the photodiode (PD) cannot receive all the reflected light but only a part of the reflected light, which affects the quality of the PPG signal. In 2016, Mohamed et al. proposed a 4-LED PPG sensor (Figure 4f) to help the photodiode (PD) receive all the reflected light [44]. MCU can control the current of four LEDs by changing the bias resistance value through the controllable switch shown in Figure 3f. A comparison of the situation using a single LED and four LEDs shows that the 4-LED structure has a higher signal strength with the same bias current. With the same signal strength, the 4-LED structure can save 30% of power consumption. At the same time, an adaptive threshold peak detection algorithm is developed to identify the peak value of PPG signals. Even for PPG signals with large amplitude changes and signals with a high baseline drift caused by MA, the error is less than 10.34%, which effectively reduces the interference of MA. Therefore, increasing the number of LEDs is a direction for optimizing PPG sensors in the future, but more research is still needed to prove the feasibility of multi-LED structures.

With the rapid development of wearable devices, how to reduce the size and weight of components while improving the integration of sensors has always been a challenge in the development of wearable devices. To reduce wired electrical connections and increase the integration of the system, in 2017, Ruedi et al. proposed a collaborative sensor to optimize the co-design of hardware and software [58]. An ASIC was integrated into the 180 nm process to measure ECG and PPG at 50 kHz. The collaborative sensors in this system were independent, meaning that each sensor has its electronics and power supply, which also provides additional degrees of freedom for the sensors. Because the current is returned through the body, the collaborative sensor requires only one electrical connection between each sensor, which is simpler than the traditional strategy, reducing the wired electrical connection and increasing the integration of the system.

**Figure 5:**
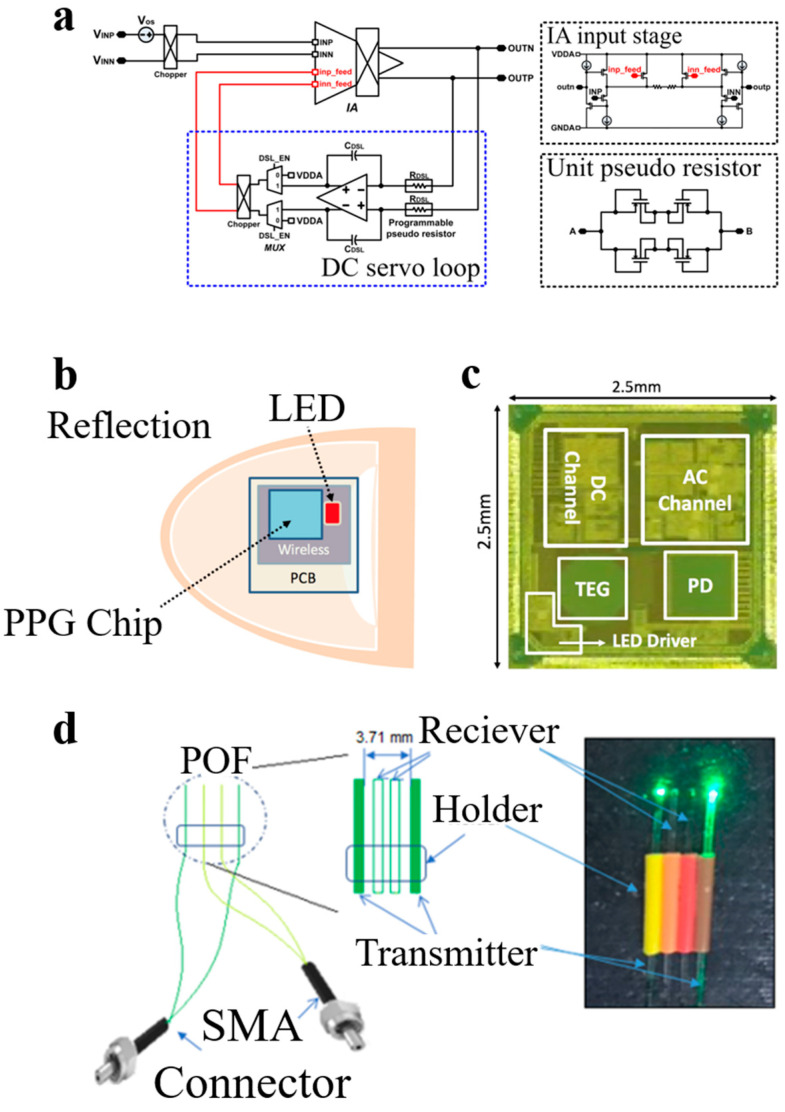
(**a**) DC servo loop; Reproduced with the permission of [59]. (**b**) Trans-nail pulse-wave monitoring system in reflection mode; (**c**) Microphotograph of the fabricated chip; Reproduced with the permission of [61]. (**d**) POF PPG probe. Reproduced with the permission of [62].

In 2018, Kim et al. designed a DC servo loop (Figure 5a) to eliminate the Deflection Coefficient (DC) deviation of PPG signals [59], which eliminated the DC deviation by giving negative feedback to the input level. The proposed IC was manufactured using the 0.18 µm CMOS process. The measured input reference current noise of the PPG readout channel was 0.122 nA/Hz, and the bandwidth was 0.5 to 100 Hz. However, in the PPG sensor without a DC servo loop [60], the reference current noise was 0.026 nA/Hz, indicating that the introduction of a DC servo loop has a good effect on eliminating DC deviation, further ensuring the accuracy of the extraction of the PPG signal peak, feature point, and other operations.

In 2018, Lee et al. designed a sensor that can measure PPG signals on the fingernail (Figure 5b) [61]. The LED circuit driver includes a current driver, a pulse-width modulator (PWM), and a ring oscillator. PWM allows the duty cycle ratio of an LED to vary from 8.26% to 99.75%. The addition of a ring oscillator enables the circuit to generate an ultra-low oscillation frequency of 0.17 Hz. The sensor was manufactured by the 0.18 μm CMOS technology (Figure 5c), with a circuit area of 6.25 mm^2^; the PPG sensor can be attached to the nail in the form of a patch with excellent portability and comfort. The collected PPG signals are of high quality, which is conducive to subsequent operations such as the feature point extraction of PPG signals. This patch-type PPG sensor has the characteristics of high integration, portability, and so on.

In 2019, Zaki et al. developed a PPG sensor based on plastic fiber optics (POF) [62], taking advantage of fiber optics’ ability to transmit and receive light, as an alternative to larger and unwearable commercial sensors. As shown in Figure 5d, the PPG sensor contains four plastic fibers with a diameter of 500 ± 23 µm, made of polymethyl methacrylate (PMMA). The tip of the fiber is cut at 45 degrees to transmit light to the skin and receive the reflected light. The optical fibers on the far left and far right act as emitters, emitting green light to the skin. The two optical fibers between the transmitters receive reflected light from the skin. The overall size of the PPG sensor is small. The distance between the emitters is 3.71 mm, and the distance between each optical fiber is 1.27 mm. SMA connectors are used to couple the emitter fiber to the light source and the receiver fiber to the phototransistor. The output of the phototransistor is connected to the RC low-pass filter with a cutoff frequency of 3.8 Hz (R = 10 kΩ, C = 2.2 µF), and high-quality PPG signals can be collected at the finger, wrist, and foot. At the same time, the non-toxicity and biochemical inertia of plastic fibers allow them to be safely applied to human skin and easily combined with fabrics to form smart fabrics with super wearable properties. Vilarinho et al. integrated a plantar pressure monitoring system based on fiber Bragg grating (FBG) technology into the insole [63]. The smart insole can monitor the patient’s plantar pressure distribution during various gaits. Therefore, optical fiber sensors may be a candidate form of PPG sensors, and combining sensors with fabrics to produce smart fabrics also presents a promising method.

**Figure 6:**
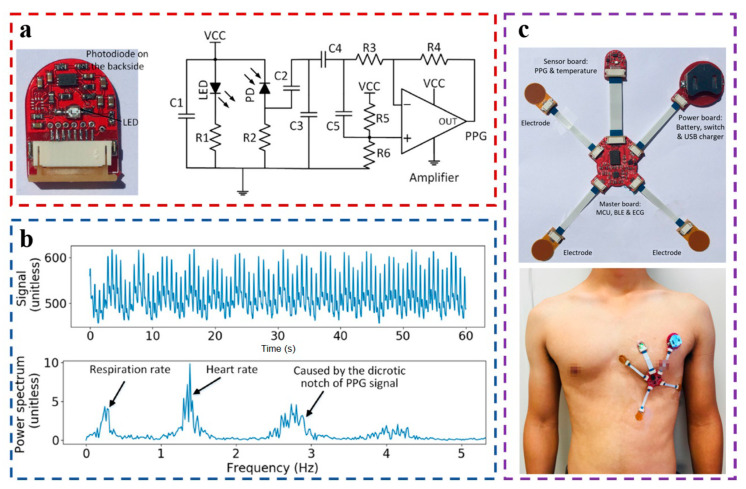
(**a**) PPG sensor (20 mm × 16 mm) and circuit diagram of the active low-pass filter; (**b**) Signals collected by the PPG sensor and spectrum analysis of the PPG signals after removing the dc component; (**c**) Rigid-flex design of the sensor patch for health monitoring. Reproduced with the permission of [64]. Copyright 2020, IEEE.

In 2020, Wu et al. proposed a PPG sensor for detecting PPG signals in the chest, taking advantage of the small distortion of PPG signals due to chest movement [64]. In Figure 6a, the skin tissue is illuminated by a green LED. The reflected light is collected by a surface-mounted photodiode (APD9008 from Avago). As shown in Figure 6b, the output signal of PD is processed by an active low-pass filter, which will remove high-frequency noise and increase the amplitude of the PPG signal. The power consumption of the sensor is low (18.03 mW), and the average current is 5.46 mA. With a 120 mAh micro battery, it can work for 21.98 h. During the experiment, it was found that the chest PPG sensor could obtain high-quality PPG signals (Figure 6c). The fast Fourier transform (FFT) was applied to the spectrum analysis of PPG data, and the parameters such as the HR and respiratory rate (RR) could also be calculated. As shown in Figure 6c, the author also uses flat, flexible cables to connect the chest PPG sensor, the ECG sensor, and the center board for data transmission and other modules, forming a wearable device for detecting physiological signals in the chest, which can accurately detect physiological parameters such as PPG, ECG, and PAT. At the same time, the small wearable device located on the chest not only has the characteristics of low-motion artifacts but also has better freedom and portability, which is conducive to improving the comfort of the wearable device. Therefore, the chest can be considered an option for the use of wearable devices. The chest patch-type physiological signal monitoring system can be developed to improve the signal quality and provide good portability and comfort.

**Figure 7:**
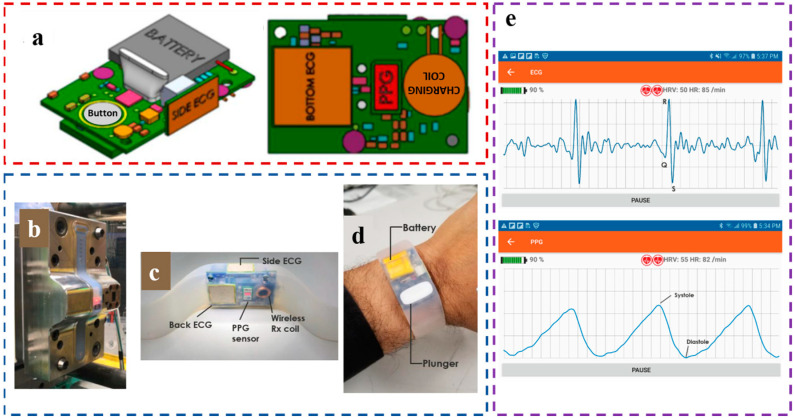
(**a**) Top and bottom views of the mechanical CAD model showing component placement following complete assembly; (**b**) An over-molded PCBA in clear silicone; (**c**) Some visible components labeled; (**d**) Wearable wristband; (**e**) App UI showing the live ECG signal and PPG signal. Reproduced with permission from [65].

In 2021, Bhagat et al. also used a PPG sensor with four LEDs to measure PPG signals (SFH7072 from Osram) [65], which was similar to the PPG sensor designed by Mohamed et al. [44]. This study presents a front-end that integrates both PPG and ECG, but we focus on its PPG sensors here. The optical PPG photodiode, Ag/AgCl ECG electrode, Nordic nRF52832 microcontroller, and other modules were integrated onto the same circuit board with the size of 30 × 22.8 × 10.8 mm^3^ (Figure 7a). It also uses a three-axis accelerometer to detect body movements (stationary, walking, running) and transmits the data to a smartphone via Bluetooth. To prepare a device with a more wearable performance, the PCB was put into the wristband built with a kind of low-temperature liquid silicone rubber (Figure 7b−c), avoiding possible damage to the electronic devices due to the high temperature during the traditional silicone resin curing. The device is worn on the wrist by the user (Figure 7d). Under different body movements, the ECG signal measured has clear QRS wave groups, and the PPG signal is very smooth and has obvious peaks and trappings (Figure 7e), which is conducive to the extraction of feature points to calculate PTT. It proves once again that the PPG sensor with a 4-LED structure can eliminate motion artifacts and shows higher accuracy.

**Figure 8:**
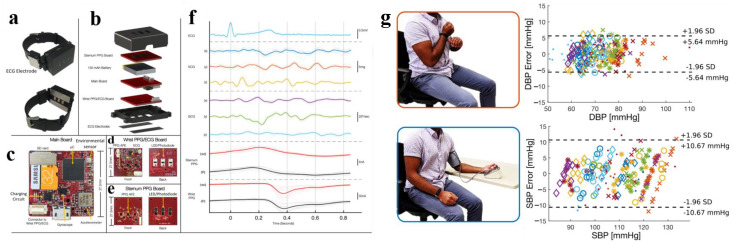
(**a**) The shape of the watch; (**b**) The components of the watch; (**c**) Main board; (**d**) The wrist PPG/ECG board; (**e**) Sternum PPG board; (**f**) Ensemble averaged waveforms from a single 30 s recording; (**g**) Experimental setup and data analysis. Reproduced with the permission of [66]. Copyright 2021, IEEE.

With the continuous rise in people’s demand for non-invasive monitoring at home, much research has been conducted to develop wristbands, patches, earwear, glasses, and other monitoring devices, but they are still present within the laboratory. In 2021, Ganti et al. created a novel and unique combination of sensors in a powerful multi-mode, non-invasive, and fully wearable wristband device to provide a practical continuous BP monitoring device [66]. This study presents a front-end that integrates both PPG and ECG; all sensors are integrated into a small table body (27.5 × 27.5 × 16.1 mm^3^, Figure 8a,b), including three circuit boards (PCBs) and a 150 mAh lithium-ion battery. As shown in Figure 8c, the mainboard is a BME280 with a small package size (2.5 × 2.5 mm^2^), a low current consumption (3.6 µA), and a low-noise floor pressure sensor (0.2 Pa RMS). As shown in Figure 8d–e, a microcontroller, ATSAM4LS8B, with a large amount of storage (512 kBytes Flash, 64 kBytes RAM), a large number of peripheral options (48 GPIO, 4 USART), and ultra-low power consumption (1.5 µA) is selected. Green LED, red LED, infrared LED, and two photodiodes (PD) SFH7072 are selected to measure the PPG signal. A low-noise (lower limit 25 µg/√Hz), high-resolution (0.003 mV/bit) accelerometer, ADXL355, is selected to measure the SCG. The BMG250 with output noise as low as 0.007°/s/√Hz was used to measure GCG signals, and the ADS1291 with low noise (8 µVpp) and high-resolution ADC (24 bit) was used to measure the ECG signals. In use, PPG, ECG, and other signals can be accurately measured (Figure 8e), and PPT is derived to calculate BP. After comparing with the data of the cuff sphygmomanometer (Figure 8f), the mean absolute deviation (MAD) of DBP and SBP was found to be 2.24 mmHg and 4.03 mmHg, respectively. This meets the IEEE standard (<5 mmHg), so it can reliably and conveniently track the natural diurnal variation of BP at home. At present, although there are still many problems in the PPT model, the calibration method, the signal processing algorithm, multi-sensor fusion, and other aspects, this study verifies the feasibility of the application of wearable BP monitoring devices out of the lab and lays a foundation for the development of wearable devices that are suitable for use at home in the future. In other words, wearables of the future will be designed to fit in the home and monitor physiology during the day. Meanwhile, numerous studies have shown that the wristband monitoring device has good potential for development. Not only can they collect effective signals at the wrist, but they also have better portability and comfort, which indicates that the wristband may become the mainstream of the BP monitoring devices.

**Figure 9:**
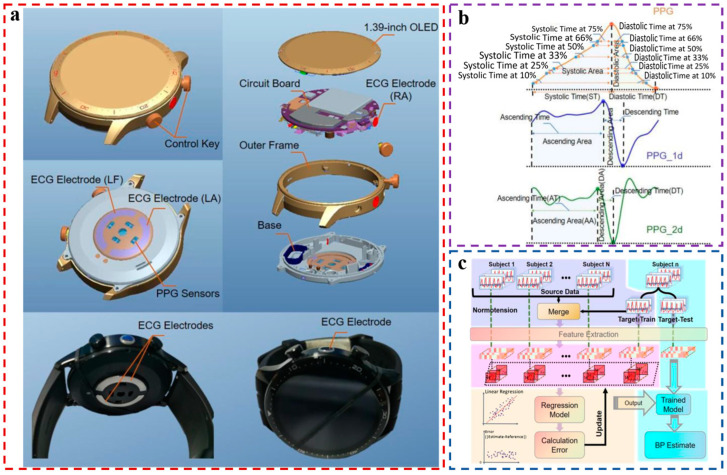
(**a**) Smartwatch that can collect ECG and PPG signals synchronously; (**b**) Time-domain features extracted from a single pulse period; (**c**) Process of the personal calibration mode based on transfer learning. Reproduced with the permission of [8]. Copyright 2022, Elsevier.

This idea that continuous BP monitoring devices will move towards the wristband is supported by recent research. In 2022, He et al. designed an application-level smartwatch capable of gathering both ECG and PPG signals [8], and this study presented a front-end that integrates both PPG and ECG. As shown in Figure 9a, the main control chip of the smartwatch is STM32U575, which supports a clear display on the graphical user interface. The PPG sensor is integrated at the bottom of the circuit board and transmits and receives optical signals through the four holes at the bottom. The ECG signals are collected using dual leads. The two ring electrodes are embedded in the bottom of the smartwatch. The ring electrodes in the right and left areas are the positive electrode (LA) and the uncorrelated electrode (LF), respectively. As shown in Figure 9b, after ECG and PPG signals are collected, feature points are extracted from PPG signals for model training, and the feature recursive elimination method is used to sort the features. As shown in Figure 9c, a personal calibration model based on transfer learning is established. The first 1–10 effective physiological data and BP data of a subject are taken as the training set of the target region, the remaining effective data of the subject are taken as the test set of the target region, and all the data of the other subjects are taken as the source domain. The simple linear regression algorithm is used to reduce the calculation amount of the model. Meanwhile, the TrAdaBoost algorithm of transfer learning constantly adjusts the sample weight in the training process [67]. In the training process, the weight of the sample data in the source domain that contradicted the data in the target domain during the training process was continuously reduced, and the BP of the remaining data of the subjects was estimated. The estimated accuracy of DBP and SBP was 0.02 ± 5.94 mmHg and 0.3 ± 7.69 mmHg, respectively, which meets the standard of AAMI. The smartwatch in this paper is very similar to the sports watch on the market, with super practicality to meet the needs of daily applications.

**Figure 10:**
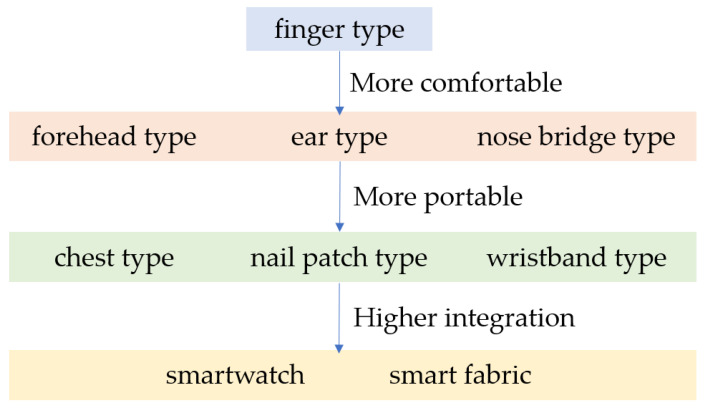
The development of PPG technology.

In the past 15 years, PPG technology has developed rapidly, and more and more researchers are paying attention to the field of wearable medical devices. As shown in Figure 10, in terms of the design of the PPG sensor, from the initial finger type to forehead type, ear type, nose bridge type, and then from chest type, nail patch type to wristband type, the PPG technology in the comfort, portability, and other aspects has continuous improvement. In terms of application scenarios of PPG sensor, from placing the sensor on the finger, forehead, nose bridge, earlobe, wrist, and other body parts to integrating multiple sensors into the smart fabric and smartwatch, it provides a scene that is easier to promote its practical application, among which smartwatch is regarded as one of the most potential development directions. However, there are still many problems to be solved for PPG sensors, such as motion artifacts, which are a major difficulty in achieving the stable performance of PPG sensors in daily life. More research needs to focus on the impact of posture changes on PPG signals. In addition, the application needs of the home level also put forward higher requirements for the lightness and integration of wearable devices.

### 2.4. ECG Sensors

The principle of ECG technology is as follows. During systole and diastole, the heart generates the bioelectric current that is conducted to the surface of the body by the conductive tissue and fluid surrounding the heart. ECG electrodes are placed at specific points on the surface of the body to detect potential caused by the heart’s bioelectric current.

In 2008, Espina et al. monitored ECG signals using a standard medical electrode with the single lead ECG patch on the chest [51]. The standard medical electrode ensured the mechanical stability and electrical coupling of the skin–electrode contact and thus was not interfered with by noise during motion. From the ECG signal of the subjects, the R-peak both at rest and during movement was able to be extracted. However, the traditional standard ECG electrode is a usually wet electrode, which is complicated to use and requires medical staff to help clean the skin of the subjects and apply the conductive gel. The gel may cause skin allergic reactions after long-term use, and signal quality attenuation will happen due to the evaporation of water in the wet electrode. Therefore, designing a convenient ECG electrode with better biocompatibility and long-term endurance is an urgent problem to be solved.

In 2010, Xu et al. designed and fabricated a novel micromachined physiological recording electrodes with hollow microneedles [53]; the microneedle array is made of heavily doped silicon, which provides excellent electrical conductivity. The microneedle can directly penetrate the outer surface of the skin, reducing the electrode–skin–electrode impedance and eliminating the need for skin cleaning. The ECG signal is extracted to facilitate the extraction of the R-peak and the calculation of the heart rate.

**Figure 11:**
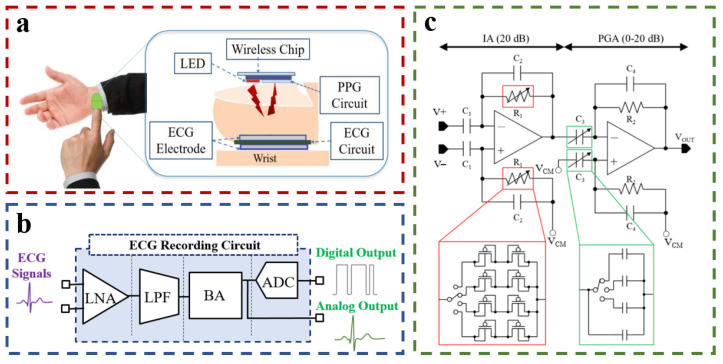
(**a**) Conceptual drawing of equipment; (**b**) Block diagram of the ECG circuit; (**c**) Schematic diagram of LNA. Reproduced with the permission of [31]. Copyright 2018, IEEE.

When using an ECG device to measure ECG signals, a ground electrode is usually required as a reference (usually placed on the right leg). Other electrodes are often placed far away from the right leg, resulting in a decrease in the integration of the system. In 2018, Lee et al. designed a small device capable of measuring ECG signals at the fingertips (Figure 11a) [31]. The circuit framework is shown in Figure 11b, which is mainly composed of a low-noise amplifier (LNA), low-pass filter (LPF), and buffer amplifier (BA). The LNA is a combination of an instrumentation amplifier (IA) and a programmable gain amplifier (PGA). Operational transconductance amplifiers (OTA) are used as the active circuit element (Figure 11c). The maximum gain that the LNA is designed to achieve is 40 dB. The ECG signal is passed through a low-pass filter (LPF) with adjustable cutoff frequencies of 0.2−10 kHz. The signal is amplified by a buffer amplifier (BA) with a variable gain of 0−20 dB. To avoid the use of a grounded electrode, a stable voltage of 0.9 V (VCM) was provided to enable the ECG signal acquisition at the fingertips using only two electrodes. To achieve low cutoff frequencies (such as 1 Hz), MOSFET resistors are used to provide resistance in the range of gigaohms while maintaining a small active area (2.53 mm^2^). This approach makes the ECG device more compact and integrated.

**Figure 12:**
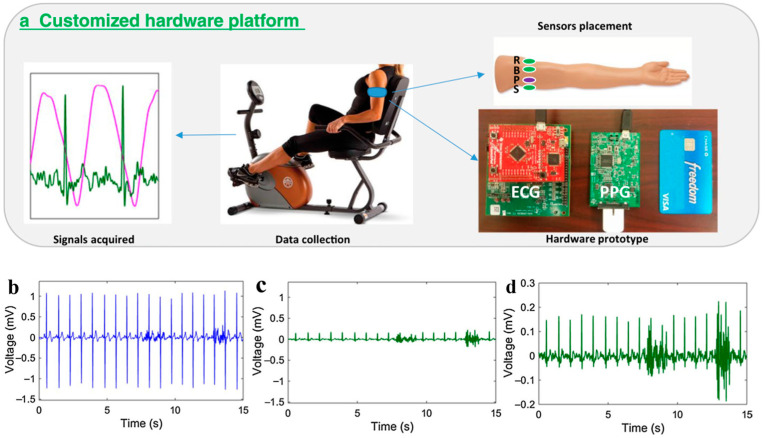
(**a**) Customized hardware platform; (**b**) chest-ECG; (**c**,**d**) arm-ECG. Reproduced with the permission of [70].

In addition to the ease of the use and integration of ECG devices, wearable comfort has always been a concern. To obtain a distinct ECG signal, the ECG sensor is usually placed on the chest [68], but the chest strap needs to be placed around the body to hold the sensor in place, causing discomfort to the user and sweating during prolonged use in hot weather. To avoid the use of chest straps, many researchers have also adopted the setting of a dual-wrist ECG plus finger PPG [69]. However, due to the long distance between the two wrists, additional leads are required to collect the potential difference between the skin of the two wrists, which is very inconvenient in daily use. At the same time, the existence of leads is not conducive to the sleepiness of users, and the effects of night monitoring are affected. In 2017, Zhang et al. integrated both ECG and PPG sensors into a single armband (Figure 12a) [70], avoiding additional wires to provide super wearability. The positions of the ECG and PPG sensors on the upper left arm are shown on the right of Figure 12a, where the circles labeled R/B/S represent the reference/offset/signal electrodes used in the ECG signal measurement, and the circles labeled P correspond to the reflective PPG sensors. However, due to the proximity of the two ECG electrodes in the armband, it is difficult to extract a high-intensity ECG signal. Therefore, by creating a customized hardware prototype and placing the two ECG electrodes on the upper and lower left upper arm to maximize the distance between the two electrodes, they successfully obtained a weak single-arm ECG signal with discernable peaks (Figure 12c,d). The signal strength is 10% of the chest ECG signal strength (Figure 12b). This study provides a new idea for the selection of application parts of wearable devices on the human body. Sensors can be integrated into commonly used clothing, such as healthcare products such as arm straps, knee pads, and wrist guards, which can not only greatly improve the wearable performance of devices but also bring great comfort and freedom to users.

When using the ECG signal and PPG signal to calculate PTT/PAT, the R peak of the ECG signal needs to be detected, and the accuracy of BP estimation largely depends on the accuracy of R peak detection. In 2019, Mishra et al. adopted the Pan–Tompkins algorithm to detect the R peak value [71]. First, the QRS energy at the output of the filter is optimized by a 5–15 Hz band pass filter (BPF) to provide the highest slope information of the signal. Secondly, the normalized signal is fed back to the square block to obtain the positive output at each moment, and the width of the QRS wave group (150 ms × fs) is captured by a moving average filter (MAF). Finally, the MAF and differentiator block output signals will be used for adaptive dual thresholding to detect R peaks. The method used detects the R-peak with a false detection rate (FDR) of 1.289% and an RMSE of 9.98% for SBP using a linear model based on PTT, which can accurately detect arrhythmias and estimate BP.

**Figure 13:**
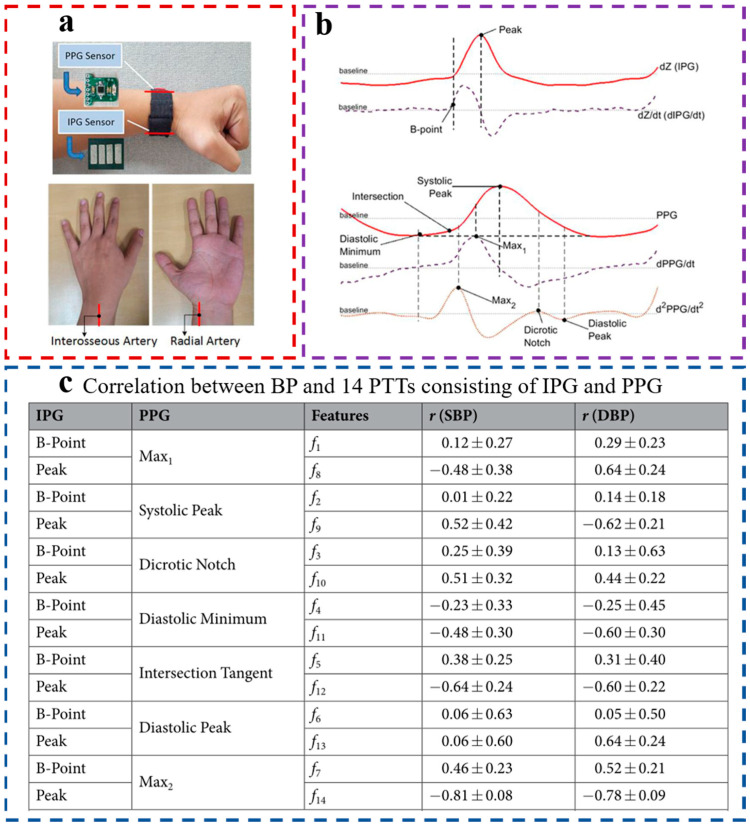
(**a**) Multimodal biosensor with the IPG signal and PPG signal; (**b**) Overall detected reference points; (**c**) Different PTT feature correlation coefficient values with SBP and DBP values. Reproduced with the permission of [72].

Based on many studies, we found that the PTT derived from ECG and PPG signals has a high correlation with SBP but a low correlation with DBP, which makes it difficult to improve the accuracy of DBP estimation. Therefore, it is necessary to find an indicator with a high correlation with both SBP and DBP. In 2019, Rachim et al. proposed a multi-mode wrist biosensor (Figure 13a) [72], which uses an impedance volume signal (IPG) instead of an ECG signal. Various characteristics of IPG and PPG were used to derive a variety of PTT for BP estimation, and finally, the best PTT characteristics were determined. As shown in Figure 13b, the zero-crossing position before the peak value and the maximum derivative of IPG was extracted, seven feature points (such as the PPG contraction peak, PPG relaxation peak, etc.) were extracted from the first derivative of PPG and the second derivative of PPG, thus forming 14 kinds of PTT, and the exponential function was used to estimate BP. The correlation between the PTT and BP formed by each IPG feature and PPG feature is shown in Figure 13c; PPT14 (f14) has the highest correlation with BP, the correlation with SBP is 0.81 ± 0.08, and the correlation with DBP is 0.78 ± 0.09. PPT14 is the interval between the peak value of IPG and the peak value of the second derivative of PPG. After the same exponential-type function was used to estimate BP. The PTT derived from ECG and PPG was highly correlated with SBP (−0.83 ± 0.17) but less correlated with DBP (−0.63 ± 0.14). Therefore, the PTT derived from IPG and PPG had a higher correlation with BP; this is helpful in improving the accuracy of BP estimation. This study also shows that the extraction of PPT does not have to just rely on PPG and ECG; more physiological signals can be mined to derive PTT, and more feature points can be reasonably extracted from various physiological signals to optimize the calculation of PTT. However, most current studies derive PTT based on PPG and ECG; few studies use other signals to derive PTT. This may be a way to improve the correlation between PTT and BP and the accuracy of BP estimation in the future to try to extract PTT by combining it with other physiological signals.

Conventional ECG signals are collected using Ag-AgCl electrodes, but as the user moves, increasing baseline drift and motion artifact noise make them unsuitable for wearable monitoring. In 2020, Pandian et al. developed a customized ECG sensor using silicone rubber and sterling silver fillers [73]. It is worn as a belt and obtains an ECG through a standard double-lead ECG device. The ECG electrode has a volume resistivity of 0.005/m and a thickness of 2.5 ± 0.25 mm. The ECG signals were acquired without any noise, baseline drift, or motion artifacts. The accuracy of RR intervals during standing and walking was 0.0038 s and 0.0039 s, the accuracy of QRS duration was 0.0035 s and 0.005 s, and the accuracy of QT intervals was 0.0212 s and 0.0282 s, respectively. According to the Bland–Altman plot, the RR interval, QRS duration, and QT interval during resting and walking were all within the ± standard deviation (S.D.) range, indicating the good stability of the ECG signals recorded during rest and walking. Meanwhile, ECG sensors, PPG sensors, etc. are integrated into the fabric and connected to the hardware for data acquisition and processing through wires woven or stitched into the fabric. An intelligent vest system for the remote monitoring of physiological parameters is made. The wire integrated into the fabric has a resistance of 0.3 Ω/m, a thickness of 0.19 mm, a tensile strength of 30 N/mm^2^, a temperature range of −65 °C to +150 °C, and a specification of 30 AWG. The clinical verification of the system compared with standard measurement methods showed that the smart vest system meets the CE specification and can be used for daily wearable physiological monitoring (ECG, PPG, HR, body temperature, etc.). In this study, sensors are placed on specific body parts and integrated into vests in a way that is in good contact with the skin surface, proving the feasibility of integrating various sensors into fabrics to prepare smart fabrics once again. Users will not be bound and can move freely. Such smart clothing with super wearable performance may be a major development direction of wearable devices in the future. With the continuous development of ECG sensor technology, the shortcomings of traditional ECG electrodes such as the cumbersome steps, the inability to be reused, them easily causing allergies of the skin, etc. have been solved, but the integration and comfort of the ECG sensor still need to be improved. Conventional Ag-AgCl electrodes increase baseline drift and motion artifact noise. Novel materials such as silicone rubber and sterling silver fillings [73], higher-dimensional carbon materials such as graphene and carbon nanotubes [74], the combination of fiber and graphene [75], and degradable materials such as polyvinyl alcohol [76], polyhydroxyalkanoates [77], etc. should be used to prepare ECG electrodes to ensure more accurate ECG signal extraction. The accuracy of BP estimation largely depends on R-peak detection. Therefore, it is still necessary to develop a more robust and accurate R-peak detection algorithm.

### 2.5. PTT-Based BP Estimation Model

In addition to PPT/PAT, there are many other physiological indicators, such as heart rate, height, weight, age, BMI, etc. Different physiological indicators can reflect different physiological information. Therefore, combining various physiological indicators can reflect human physiological conditions more comprehensively.

**Figure 14:**
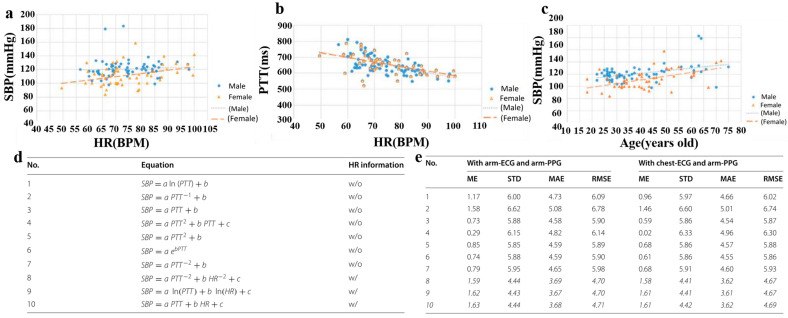
(**a**) Relationship between HR and BP; (**b**) Relationship between HR and PTT; (**c**) Relationship between gender, age, and SBP; Reproduced with the permission of [78]. Copyright 2016, IEEE. (**d**) Ten BP models for comparative analysis; (**e**) The testing performance of ten SBP models. Reproduced with the permission of [70].

In 2016, Hsiao et al. added different physiological parameters to the BP estimation equation (Table 1) to select the best parameter combination [78]. As can be observed in Table 1, Hsiao et al. tried to add various physiological parameters to the equation. The experimental results show that the error decreases when one more physiological parameter is added to the equation. On the one hand, it can be seen from Figure 14a,b that HR has a greater impact on PTT than on SBP. For both men and women, the data in Figure 14b tend to have a higher correlation than those in Figure 14a. Therefore, it is necessary to include HR in BP estimation to correct PTT. On the other hand, it can be seen from Figure 14c that SBP is proportional to age, that is, BP increases with age, but the BP of women is generally lower than that of men. Therefore, different estimation equations should be provided for different genders. The final experimental results show that the error of SBP is 6.9 mmHg ± 8.6 mmHg, which does not meet the AAMI standard (5 mmHg ± 8 mmHg) but was close enough to indicate the feasibility of introducing physiological parameters other than PTT into the BP estimation equation.

In 2017, Zhang et al. applied the PTT&HR–SBP model to SBP estimation (Figure 14d) and compared it with many PTT–SBP models [70], proving the necessity of introducing HR information into the BP regression model. As shown in Figure 14e, the performance of seven PTT–SBP models is worse than that of the PTT&HR–SBP model. Even the worst-performing Model 10 in the PTT&HR–SBP model has ME ± STD, MAE, and RMSE values of 1.63 ± 4.44, 3.68, and 4.71, respectively. Compared with Model 1, STD, MAE, and RMSE are reduced by 35.1%, 28.5%, and 29.3%, respectively, which meet the AAMI standard. It can also be seen from Figure 14e that the PTT&HR–SBP model is more stable than the PTT–SBP model. Therefore, introducing HR and other information into the BP regression model will help improve the accuracy and robustness of the BP calculation model.

**Figure 15:**
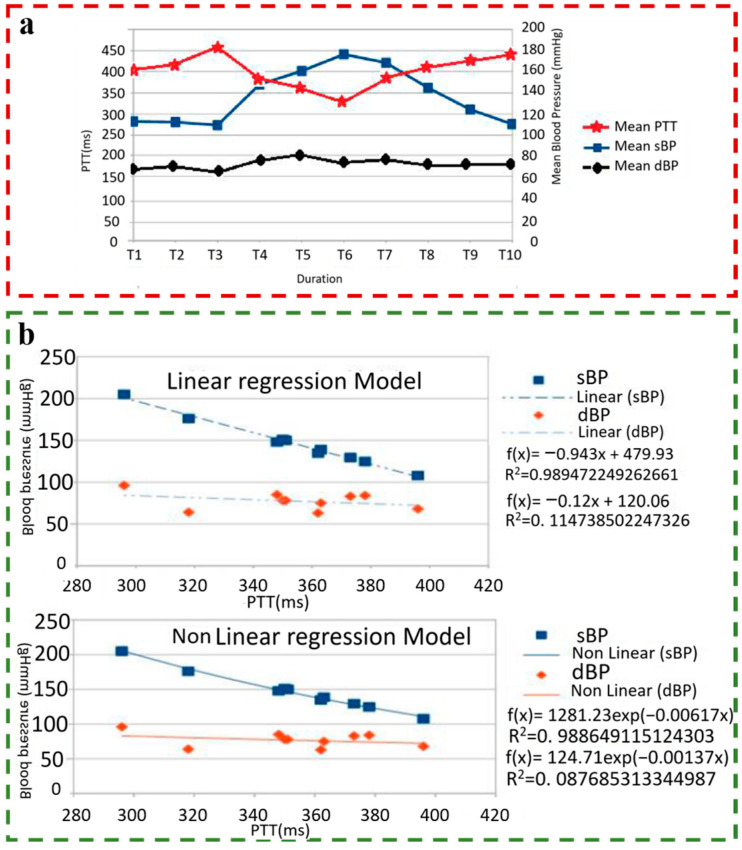
(**a**) Mean PTT, Mean SBP, and Mean DBP from subjects; (**b**) Best-fit curves for patients using the linear regression model and non-linear regression model. Reproduced with the permission of [79]. Copyright 2017, IEEE.

In 2017, Aboughaly et al. compared the accuracy of linear and nonlinear BP regression models on the same dataset, in which the nonlinear BP regression model selected an exponential-type function [79]. After calculation, in the linear regression model, PTT and SBP have strong correlation characteristics (Figure 15a), with 0.92~0.99, but the correlation between PTT and DBP is very weak. As shown in Figure 15b, the linear model assumes negative values of PTT for high BP values and vice versa, while the nonlinear model provides a more realistic prediction for the relationship between PPT and BP. Therefore, the nonlinear model may be the future BP estimation equation’s main form.

In 2017, Lu et al. estimated BP using PTT and a large number of features extracted from PPG waves based on statistical learning and predictive analysis [80]. Three kinds of PTT approximations were used in the prediction model, including the time between the start of the QRS wave group (ECG), the start of the PPG wave, the maximum rise point of the PPG wave, the peak value of the PPG wave, and features extracted from the first and second derivatives of the PPG signal. Finally, the predicted BP met the AAMI and IEEE standards. It is proved that the PPG signal and its derivative data contain rich physiological information to a certain extent, indicating that it is necessary to carry out deeper information mining of the PPG signal; reasonable feature extraction of the PPG signal will be conducive to BP estimation.

**Figure 16:**
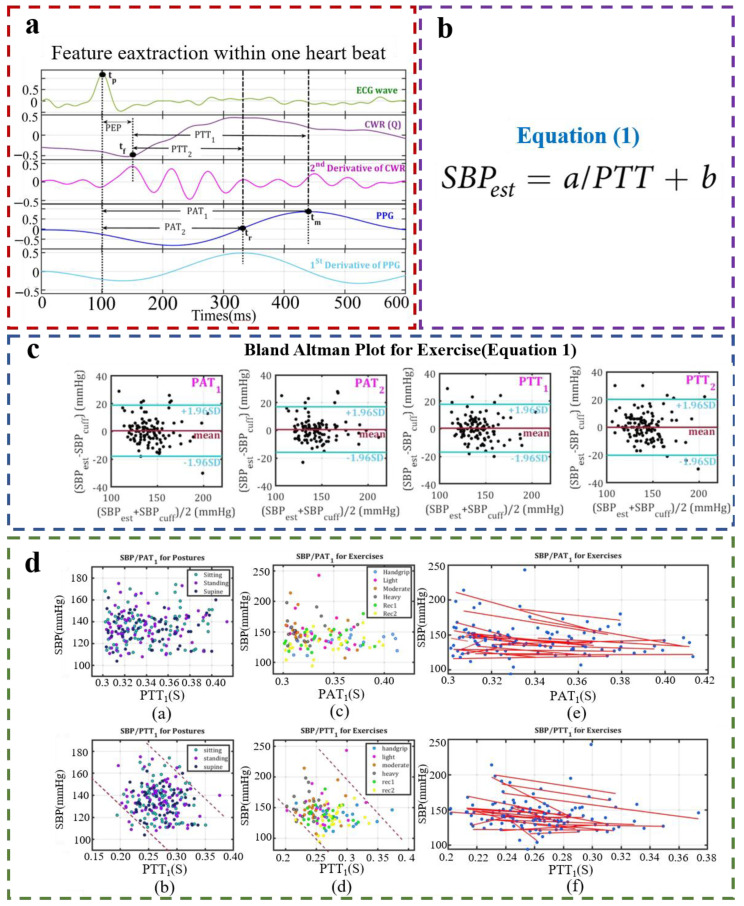
(**a**) Samples of ECG, CWR, PPG, the second derivative of CWRs, and the first derivative of PPGs for a subject in a standing position; (**b**) BP estimation equation; (**c**) Bland–Altman plots for exercise tasks using Equation (1); (**d**) Fitted curves of PTT and PAT to BP. Reproduced with the permission of [81].

Although PTT and PAT are two completely different concepts, some scholars regard PAT and PTT as the same index, without considering which is more suitable for BP estimation. Therefore, the accuracy of these two methods should be evaluated and compared to determine whether the addition of PEP can improve the accuracy. In 2019, Ebrahim et al. compared the PTT and PAT methods [81]. Instead of using only the PPG sensor and ECG sensor to measure PTT, the on-body continuous wave radar (CWR) was added to measure the pre-ejection PEP. PTT was calculated and combined with PAT (Figure 16a), and the SBP of PTT-based/PAT-based was calculated by a mathematical model (Figure 16b). As shown in Figure 16c, it can be seen that the calculation of SBP by PTT has considerable improvement compared with that by PAT. Removing PEP from PAT improves accuracy by about 9% based on the cumulative errors calculated in the literature. Meanwhile, the author also collected data under different postures and movement conditions. Figure 16d shows the SBP results measured under three different postures, indicating that there is no clear relationship between the PAT value and SBP, while PTT and SBP remain negatively correlated despite the same trend due to posture changes. There was an inverse relationship between PTT/PAT and SBP during exercise. It can be seen that the fitted curves based on the PAT for different subjects were more dispersed than those based on PTT, while the calculated curves of PTT–SBP were more consistent with a higher linear correlation than those based on PAT–SBP. Therefore, the performance of the PTT–SBP model can be effectively improved after the removal of PEP. In the future, when using the BP calculation model based on PTT, attention should be paid to eliminating PEP to improve the accuracy of the model.

After PTT is derived using the PPG and ECG signals, BP is usually calculated by establishing a regression model, but more and more studies have shown that combining PTT with other physiological parameters can construct a more accurate and stable BP estimation model. At the same time, the PPG and ECG signal itself also contains very rich feature information (PPG first derivative, second derivative, etc.), which can play a role in optimizing the BP model and will be conducive to the estimation of BP. Therefore, it is necessary to dig deeper into the information contained in various physiological signals. The relationship between various physiological parameters and BP estimation deserves more attention to establish a multi-parameter BP regression model based on PTT.

As shown in Table 2, some details of 22 articles from 2008 to 2022 are presented. With the continuous development of physiological signal monitoring technology, on the one hand, it can be seen that the PPG and ECG measurement position has been transferred from the ear, the bridge of the nose, and other positions to the wrist, which means that the wearable systems are more integrated and the user experience is improved. On the other hand, with the continuous improvement of the BP estimation model, the accuracy of BP estimation has been continuously improved, with more study cases meeting the AAMI standard, which lays a foundation for the early realization of continuous blood pressure monitoring at the medical level. However, little literature summarizes the weight and endurance of BP monitoring equipment. More attention should be paid to reducing the weight and improving the endurance of BP monitoring equipment in the future to better meet the needs of commercialization and the market.

## 3. Discussion and Outlook

Numerous studies have shown that PTT is highly correlated with BP, and BP can be estimated noninvasively from PTT [68,83,84,85]. This non-invasive BP measurement method avoids the shortcomings of the traditional inflatable cuff scheme. It is no longer limited to intermittent and non-continuous BP measurement but facilitates the realization of continuous daily BP monitoring. With its assistance, the latest real-time information about the human vascular system will be collected, and timely diagnosis and treatment for judging the patient’s physical condition will be achieved.

In early studies, PPG signals are typically measured in the finger, earlobe, etc. [86], and ECG signals are typically measured in the chest [58]. Wired connections are required between each sensor, resulting in the low integration, low portability, and low comfort of the wearable device, which is inconvenient to the user. In recent years, continuous BP monitoring technology based on the PTT method has developed rapidly. Researchers have found that it is possible to collect PPG signals and ECG signals simultaneously in some parts of the body (wrist, chest, etc.). Therefore, the method of collecting signals from multiple parts of the body has evolved to collect multiple signals from a specific part of the body. The design of the wearable system has also gradually developed from a multi-device signal acquisition system [87] to a single-device signal acquisition system. Many modules such as the PPG and ECG sensors are integrated into the same device, such as smartphones [11,88] and smartwatches [89,90,91] (the most popular), to continuously improve the integration of wearable systems.

In the future, the wearable devices should be compact, highly integrated, and portable and have long-term endurance. The smartwatch is one promising solution at present, because it is small, portable, comfortable, and convenient to users and can extract high-quality PPG signals and ECG signals from the wrist. The small, lightweight smartwatch will not affect the user’s daily activities. However, PTT-based BP monitoring equipment still faces many challenges, especially signal monitoring under dynamic conditions. Motion artifacts have a very serious impact on PPG sensors, meaning that the user needs to be kept in a relatively static situation to monitor physiological signals. In addition, there are still many immature aspects such as the information mining of physiological signals, multi-signal fusion, the influence of posture changes on signals, etc. Our recommendations for future wearable hardware and software research are as follows:

**Optimal design of wearable devices.** Continuous BP monitoring devices usually contain hardware modules such as a PPG sensor, ECG sensor, microcontroller, power supply module, etc. [92]. During the selection of each module, attention should be paid to the selection of low-power electronic devices to improve the endurance of wearable devices on the premise of ensuring the realization of functions. During the integrated design of each module, the circuit design should be optimized, the volume of the wearable system should be reduced as much as possible, and the portability of the wearable device should be improved. At the same time, it is necessary to consider whether it is easy to integrate with commercially available wearable devices, such as smart bracelets and other electronic products. If BP monitoring devices can be integrated into commercially mature wearable devices, it will be very conducive to promoting the daily civilian application of BP monitoring devices.

**Development of a motion artifact correction algorithm.** In current BP monitoring equipment, PPG is the most affected by motion artifacts, failing in continuous BP monitoring under dynamic conditions. Many studies have shown that it is difficult to extract effective PPG signals in the state of walking or running, and even if PPG signals are extracted, it is difficult to extract feature points. A more robust detection algorithm should be developed to identify the feature points of PPG signals [82]. Meanwhile, optimizing the design of PPG sensors is also a way to reduce the influence of motion artifacts. For example, PPG sensors with a 4-LED structure have been proven to reduce the interference of motion artifacts [44]. It is very important to study the influence of posture changes on the PPG signal and develop a motion artifact correction algorithm for PPG signal extraction.

**Deeper information mining.** After the ECG and PPG signals are extracted, the peaks of both signals are detected, usually to derive the PTT. However, in addition to detecting the peaks of the two signals, deeper information mining on the signals is ignored during the following process. At present, more and more studies have confirmed that the PPG signal and its derivative data contain rich physiological information. If more characteristic information is extracted from the PPG signal and its derivative, a more accurate BP can be estimated by statistical learning and predictive analysis [80]. Combining PTT with other characteristic information to form a multiple regression model will help improve the stability and accuracy of the BP calculation model. Therefore, deeper information mining of the PPG signal is one of the development directions for improving the accuracy of BP estimation in the future.

**More diverse signal fusion.** When using PTT as an indicator to estimate BP, PTT is usually derived from ECG and PPG signals. However, a few studies have explored the possibility that IPG signals [62] or Bio-Z signals [80] are used as a substitute for ECG signals. Various characteristics of the IPG signal or Bio-Z signal and PPG were used to derive PTT for BP estimation. The PTT derived by this method was comparable to the traditional PTT derived by ECG and PPG. It was found that when using the same BP regression model, the PTT derived from the IPG signal or Bio-Z signal and the PPG have a higher correlation with BP, which is conducive to improving the accuracy of BP estimation. The extraction of PTT in combination with other physiological signals is potentially a future way to improve the accuracy of BP estimation.

## 4. Conclusions

Many advanced BP monitoring devices have been developed for the daily continuous monitoring of BP. The information summarized in this paper may help guide the design of future wearable monitoring systems for more easily and quickly achieving continuous BP monitoring with high accuracy. In this review, we introduce the recent state-of-the-art continuous BP monitoring equipment based on PTT, review the development history of the PPG sensor and ECG sensor, and analyze their measuring principles, design methods, preparation processes, and properties in detail. The possible development direction and possible challenges of BP monitoring equipment based on the PTT method are also presented. With the continuous development of wearable devices for continuous BP monitoring, it is believed that continuous BP monitoring devices with higher integration, better portability, and comfort will be proposed, and wearable systems that are more suitable for daily continuous BP monitoring will be developed soon.

## Figures and Tables

**Table 1:** Blood pressure estimation equations and errors for different parameter combinations [78].

Equation	D ± Std Dev (mmHg)
SBP = a × PTT + b × HR + k	7.33 ± 9.02
SBP = a × PTT + b × HR + c × BMI + k	6.96 ± 8.78
SBP = a × PTT + b × HR + c × AGE + k	6.82 ± 8.60
SBP = a × PTT + b × HR + c × BMI + d × AGE + k	6.72 ± 8.50

**Table 2:** Some details of the reference. N/R: not reported, Y: satisfied, N: unsatisfied, CBP: cuff blood pressure, FABP: finger arterial blood pressure.

Ref	Measuring Position	ME ± SD (mmHg)	BP Estimation Model	AAMI	Gold Standard
Jung et al. (2008) [50]	PPG: finger ECG: chest	N/R	N/R	N/R	N/R
Espina et al. (2008) [51]	PPG: finger ECG: chest	N/R	$SBP=A\frac{L}{PAT}+B$	N/R	N/R
Xu et al. (2010) [53]	PPG: finger ECG: wrist	N/R	$SBP=\frac{\rho \cdot d^{2}}{1.4}\cdot \frac{1}{PTT^{2}}+\frac{1}{0.7}\rho gh$	N/R	CBP
Franco et al. (2012) [54]	PPG: forehead ECG: chest	SBP = 0.79 ± 5.50 DBP = −3.59 ± 3.29	$SBP=\frac{\alpha }{PAT}+\beta$	Y	CBP
Winokur et al. (2012) [55]	PPG: mastoid bone ECG: mastoid bone, neck	MAP = −0.07 ± 3.64	MAP=A−B·ln(PTT)+P	N/R	FABP
Zheng et al. (2012) [56]	PPG&ECG: finger, earlobe, and nose bridge	N/R	N/R	N/R	N/R
Puke et al. (2013) [57]	PPG: chest ECG: chest	SBP = 6.91 ± 4.23	$SBP=a\cdot ln\left(\frac{1}{PTT^{2}}\right)+c$	N	CBP
Wang et al. (2015) [19]	PPG: eyebrow ECG: earlobe	N/R error is within 5%	N/R	Y	CBP
Mohamed et al. (2016) [44]	PPG: finger ECG: chest	SBP = −1.36 ± 7.51 DBP = −2.44 ± 3.49	P=a·PTT+b·HR+c	Y	CBP
Hsiao et al. (2016) [78]	PPG&ECG: wrist	The same as Table 1	The same as Table 1	N	CBP
Zhang et al. (2017) [70]	PPG&ECG: upper arm	SBP = 1.63 ± 4.44	SBP=a·PTT+b·HR+c	N	CBP
Kim et al. (2018) [59]	PPG: finger ECG: wrist	N/R	N/R	N/R	N/R
Lee et al. (2018) [61]	PPG: fingernail	N/R	N/R	N/R	N/R
Zaki et al. (2019) [62]	PPG: finger, wrist, and ankle ECG: chest	N/R	N/R	N/R	CBP
Mishra et al. (2019) [71]	PPG: wrist ECG: chest	SBP = 4.20 ± 1.66 DBP = 2.90 ± 0.90	$P=A\cdot e^{B\cdot PTT}$	Y	CBP
Ebrahim et al. (2019) [81]	PPG: earlobe ECG: chest	N/R	$SBP=\frac{a}{PTT}+b$ $SBP=\frac{a}{PTT_{1}^{2}}+\frac{b}{PTT_{2}^{2}}+c$	N/R	CBP
Wu et al. (2020) [64]	PPG&ECG: chest	N/R	BP=a·PAT+b·HR+c	N/R	CBP
Bhagat et al. (2021) [65]	PPG&ECG: wrist	N/R	N/R	N/R	N/R
Ganti et al. (2021) [66]	PPG&ECG: wrist	SBP = 4.75 ± 2.29 DBP = 2.72 ± 0.75	$BP=\frac{K_{1}}{PTT}+K_{2}$	Y	CBP
Landry et al. (2021) [82]	PPG: forehead ECG: wearable body metrics vest	N/R	N/R	Y	FABP
He et al. (2022) [8]	PPG&ECG: wrist	SBP = 0.30 ± 7.69 DBP = 0.02 ± 5.94	N/R	Y	CBP
Wang et al. (2022) [22]	PPG&ECG: wrist	SBP = −2.10 ± 7.07 DBP = 0.04 ± 7.34	N/R	Y	CBP

## Data Availability

Not applicable.
